# A giant sacrococcygeal teratoma in adult female: A case report

**DOI:** 10.1016/j.ijscr.2018.11.039

**Published:** 2018-11-22

**Authors:** Nawaf Jaber Shatnawi, Muhammad Rushdi Khammash, Abdelkarim Hussein Omari

**Affiliations:** a Department of General Surgery, Division of Vascular Surgery, King Abdulla University Hospital, Faculty of Medicine Jordan, University of Science and Technology, P.O. Box (3030), Irbid 22110, Jordan; b Department of General Surgery, King Abdulla University Hospital, Faculty of Medicine, Jordan University of Science and Technology, P.O. Box (3030), Irbid, 22110, Jordan

**Keywords:** Teratoma, Sacrococcygeal, Adult rare mass, Pelvic masses

## Abstract

**Introduction:**

Sacrococcygealteratoma is very rare in adults with only few cases reported in literature. It is a slow growing benign cystic tumor with 1–2% chance for malignant transformation and may attain a huge size causing pressure effect on pelvic and intra-abdominal organs. When grows caudally, it causes discomfort, skin excoriation and cosmetic disfigurement.

**Presentation of case:**

We present a giant, long standing Sacrococcygeal teratoma in an adult female patient which caused her significant obstructing lower gastrointestinal and urinary symptoms besides the discomfort and disfigurement of the sacral region.

**Discussion:**

Sacrococcygeal teratomas are commonly present early in life but are very rare to be seen in adults with such a huge size in such a place where it interfered with patient sitting and sleeping.

Diagnosis of our case was suspected by clinical examination, ultrasound and MRI while histopathology confirmed the diagnosis. It was completely excised along with the coccyx with primary wound closure and had a good postoperative recovery. Up till now, 18 months follow-up using clinical, biochemical and radiological assessment revealed no evidence of recurrence.

**Conclusion:**

Sacococcygeal teratoma should be considered in the differential diagnosis of large pelvic masses in adults. In the presence of an extra-corporal and deep intra pelvic extension, we recommend the posterior perineal approach for excision as it has convenient control over the mass during surgery and good cosmetic results.

## Highlights

• Sacrococcygeal Teratoma causing pressure effects on intra pelvic viscera is a very rare presentation.
• Posterior perianal approach proved to be very helpful in excising the teratoma especially when it has an extracorporeal extension.
• Despite the huge size and the clinical presentation of the mass, the histopatholgical examination confirmed its benign nature.

## 1 Introduction

Sacrococcygealteratomas (SCT) are defined as tumors containing tissues derived from two or more primitive germ cells and are the commonest fetal neoplasms with a prevalence of 1/40,000 with female to male ratio of 4:1 [1].

STC is extremely rare in adults that only few cases were reported in literature and as in infants, adult females had the predominance of 4–10 times as in males [1,2]. These tumors are usually cystic and benign with only 1–2% chance for malignant transformation in adults [2,3].

Cysts may be filled with serous fluid, mucoid, or sebaceous material and lined by true epithelium.

Altman classified SCTs into 4 types according to its position: type I, predominantly external mass with a small presacral component; type II, external mass with a intrapelvic component; type III, external mass with a pelvic and abdominal component; and type IV, internal mass with an intrapelvic and abdominal location; types II and III are usually dumb-bell shaped [4].

The clinical presentations of the teratoma are mainly due to its mass effect on the adjacent organs as the rectum, urinary bladder and pelvic vasculature causing obstructive symptoms of the affected system. Projection of the mass externally will cause disfigurement and discomfort in addition to painful skin excoriation and ulcers.

CT scan and or MRI imaging are invaluable in the diagnosis of these lesions and in delineating their origin, extent and relation to the pelvic and abdominal organs. Surgical excision is considered curative when the coccyx is removed with the mass and the histo-pathological report confirmed the benign nature of the teratoma.

Our work has been reported in line with the SCARE criteria [5].

## 2 Presentation of case

A previously well 49- year old multiparous Jordanian female who was not on any medication referred to our teaching hospital complaining of the presence of a huge sacrococcygeal mass of eight years duration. The mass had progressively increased in size from a small painless nodule at the tip of the coccyx till it became large enough to cause obvious cosmetic disfigurement of the body contour and shape. It prevented the patient from lying on her back or sitting down comfortably. For the last year, patient started to complain of increased urinary frequency and constipation with a narrow caliber fecal stream. Few weeks prior to her visit, she started feeling progressive deep abdominal and pelvic pain and noticed engorgement of her lower limbs which compelled her to seek medical help.

**Fig. 1:**
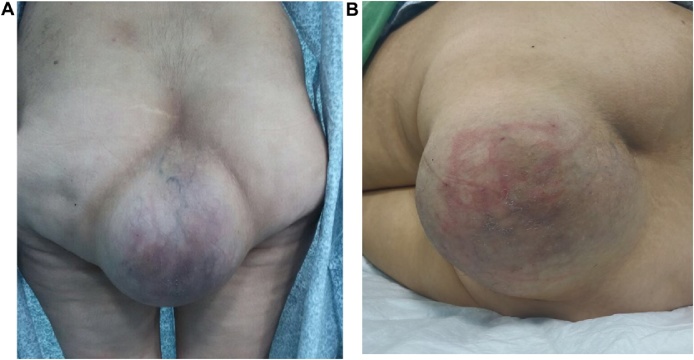
(A): Standing view showing the mass extending down the natal cleft (B): left lateral position showing a huge mass over the lower back and upper buttocks with skin excoriations.

Inspection showed a huge smooth rounded mass (20 × 20 cm) extending down the lower end of the natal cleft hanging over buttocks as shown in Fig. 1(A and B). The skin over the mass was hyperemic and excoriated. Palpation showed a slightly tender, immobile, firm mass which was inseparable from the overlying skin at the midline.

Digital rectal examination revealed a tense smooth slightly tender bulge encroaching on the posterior wall of the rectum with a normal feeling mucosa.

**Fig. 2:**
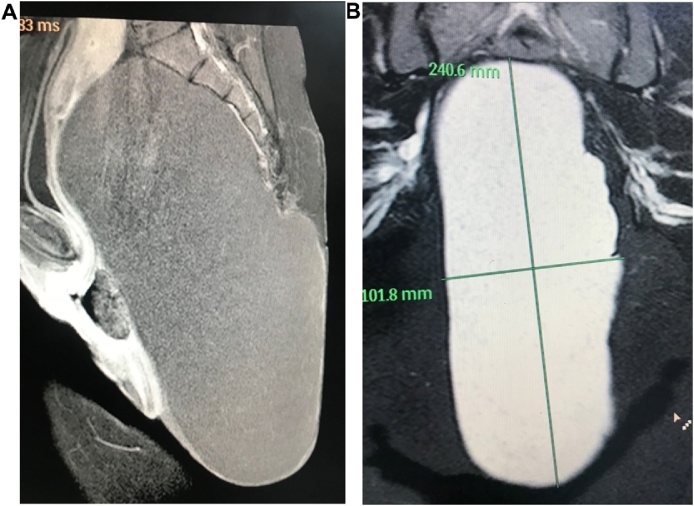
(A): MRI of the pelvis showing cyst filled with faint calcifications with clear margins except over the lower coccyx compressing the pelvic structures anteriorly and superiorly. (B): MRI showing dimensions of the cyst and relations with pelvic structures.

Magnetic resonance imaging studies (MRI) showed a well-defined encapsulated mass of 25, 15, 10 cm size. The mass was filling the presacral space, extending up the pelvis and down posterior to the rectum and anal canal compressing both and the urinary bladder with a huge extra corporal extension Fig. 2(A and B).

It appeared hypo-intense in T1 weighted images with faint calcification spots while it showed hyper intensity in T2 imaging with clear surrounding boundaries except for a close relation to the coccyx. There was no enhancement of the lesion and no invasion of the adjacent structures nor was bone erosions. MRI findings were interpreted as a Sacrococcygealteratoma, possibly originating from the coccyx. However, the differential diagnosis was going around other rare conditions as rectal duplication cysts, lipoma, liposarcoma, sacrococcygealchondromas and chordomas.

**Fig. 3:**
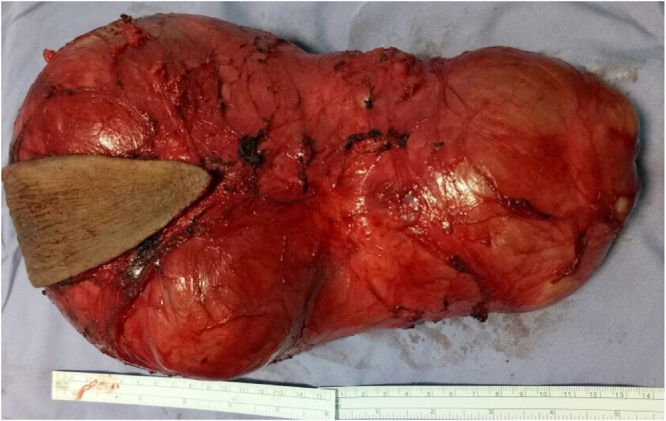
Cyst on table showing its dimensions after complete excision.

Related serum tumor markers as alpha-feto protein, human chorionic gonadotropins (HCG), carcinoembryonic Antigen (CEA), Lactate Dehydrogenase (LDH) Carcinoma Antigen (CA 125) were all normal (Fig. 3).

Fine needle aspiration was tried twice but did not yield any fluid or cells for cytological examination.

Under general anesthesia, the patient was placed in prone position and an elliptical incision was performed as in posterior perineal approach. The mass was dissected and followed up into the pelvis where it was separated from the pelvic viscera easily but when followed downwards it was found firmly attached to the coccyx. Resection of the coccyx was performed to complete the excision of the mass and the wound was closed in the midline after securing hemostasis.

**Fig. 4:**
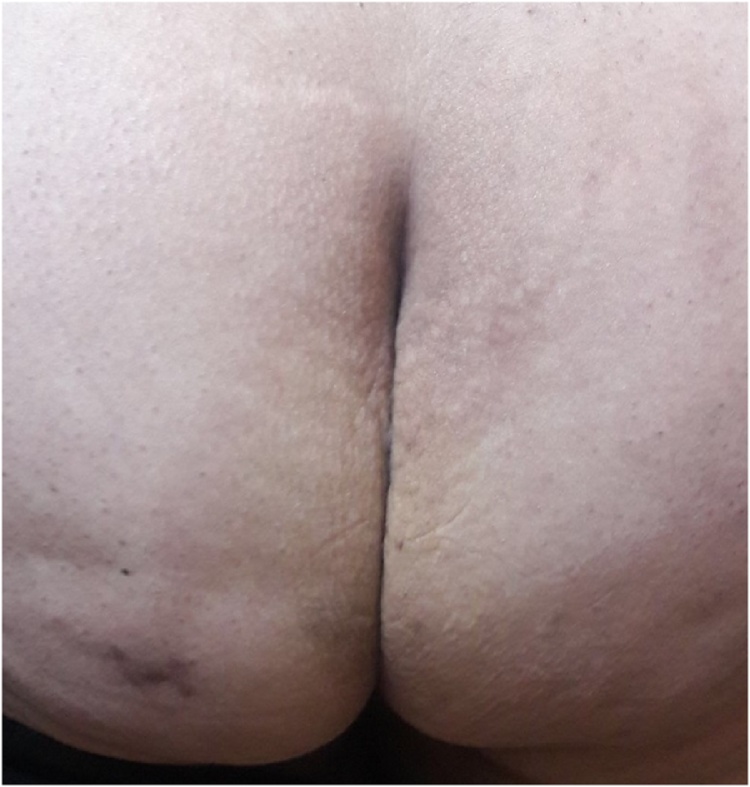
Lower back and buttocks three months after surgery.

The patient had smooth recovery with nice wound healing and cosmetic appearance (Fig. 4). The final histopathology report confirmed the diagnosis of mature teratoma.

Patient was seen regularly for 18 months by now, and a recent MRI showed no recurrence.

## 3 Discussion

Teratomas are neoplasms composed of mixed tissue elements derived from two or more germ cell layers. While the majority of teratomas present congenitally in the sacrococcygeal region, they have been identified throughout the body [1].

Sacrococcygealteratoma is one of the commonest fetal neoplasms, but is so rare in adults that only case reports and few case series were reported on reviewing the literature with lack of information of its incidence in the general population [1,2].

Both infantile and adults types of sacrococcygealteratoma tend to occur more frequently in females with a female-to-male ratio of about 4–10:1 [1,2].

Adult SCT is mostly seen as an intra-pelvic mass when compared to the neonatal variety which mostly presents as externally visible mass [3]. Teratomas are categorized pathologically as mature and well differentiated or immature and poorly differentiate that tends to undergo malignant differentiation.

In comparison to the infantile type, the incidence of malignant transformation of the adult teratomas is much lower with an incidence of about 1–2% [2,3].

As SCT mostly grows slowly within the pelvic cavity it may cause no symptoms and discovered during investigation for another intra- abdominal pathology. However, by increasing in size, it may present as deep pain and discomfort because of the mass stretching and compression effects. Larger mass could also cause symptoms secondary to its pressure effect on the pelvic viscera. Huge teratomas may extend externally to cause cosmetic and body contour disfigurement as was also noticed in our case [[1], [2], [3]].

Ultrasonic examination of the cyst usually reveals heterogenic echogenicity with cystic and solid areas [2,6]. Computed tomography (CT) and MRI are the most significant tools to characterize the mass. MRI is superior in evaluating the intra-pelvic extension and relationship to other structures because of its multiplanar imaging capability and a good soft tissue contrast [7]. In our patient; MRI examination was helpful in identifying the nature of the mass as well as its relation with the pelvic viscera and its attachment to the coccyx and the extra corporal extension.

Blood investigations might be helpful in suggesting the possibility of malignancy. This could be seen when the tumor markers as alpha fetoprotein, carcino-embryonic antigen and human gonadotrophin are elevated [2,8]. Our patient markers were within the normal range; favoring the benign nature of the mass.

Pre-operative pathological studies like trans- cutaneous or trans-rectal biopsy may not be advisable because of the risk of recurrence or dissemination in case of malignancy [7,9]. Fine needle aspiration cytology can be informative about the nature of the mass. However in our case it could not aspirate cells to report on because contents were thick.

Exploration, dissecting the mass from the surrounding viscera and excising the attached coccyx is the modality of choice to remove the cyst and its midline origin [10,11].

In our case, the histopathological examination of the specimen proved the benign nature of the cyst as a mature teratoma.

If histopathological examination showed malignant differentiation then; the possibility of recurrence could be avoided by postoperative chemotherapy and radiotherapy [12].

The operative excision is usually accomplished through laparotomy or laparoscopic exploration. However, because of the significant extrapelvic and perinealextention, we have chosen the perineal approach which proved to be helpful and ended with a good cosmetic result. This, we think, makes our case unique among other reported cases.

After repeated postoperative follow-up visits to the clinic; the patient confirmed the return of her urinary and defecation habits to normal and was happy with the satisfactory result of the healed wound.

## 4 Conclusion

Although SCT is a rare tumor in the adults, still it should be considered in the differential diagnosis of a pelvic mass especially when associated with extra-pelvis extension. Surgical excision of the tumor with coccygectomy is the treatment of choice.

Posterior perineal approach is recommended in case of extra-pelvic extension as it gives good cosmetic look in addition to convenient control over the mass during surgery.

## Conflict of interest

We declare that we have no competing interests.

## Funding source

This research received no specific grant from any funding agency in the public, commercial, or non-for-profit sectors.

## Ethical approval

Institution Review Board (IRB) of the Jordan University of Science and Technology and King Abdulla University Hospital granted the approval for all the work done in these institutions.

## Consent

Written informed consent was obtained from the patient for publication of this case report and accompanied images.

## Author contribution

1. Nawaf J Shatnawi: Took part in the surgical management, participated in the design and Coordination of the study helped to draft the manuscript and reviewed the literature.

2. Muhammad R Khammash: Participated in the interpretations of the radiological images, helped to draft and editing the manuscript.

3. Abddel karim H Omari: Took part in the surgical management, Participated in the design, studied the images and reviewed the literature.

All three authors read and approved the final manuscript.

## Registration of research studies

Not applicable.

## Guarantor

Nawaf J. Shatnawi.

## Provenance and peer review

Not commissioned externally peer reviewed.
